# Antimicrobial activity of *Buchenavia tetraphylla* against *Candida albicans* strains isolated from vaginal secretions

**DOI:** 10.1080/13880209.2017.1304427

**Published:** 2017-04-05

**Authors:** José Robson Neves Cavalcanti Filho, Tiago Fonseca Silva, Woah Queiroz Nobre, Larissa Isabela Oliveira de Souza, Cristiane Santos Silva e Silva Figueiredo, Regina Celia Bressan Queiroz de Figueiredo, Norma Buarque de Gusmão, Márcia Vanusa Silva, Luís Cláudio Nascimento da Silva, Maria Tereza dos Santos Correia

**Affiliations:** a Departamento de Bioquímica, Universidade Federal de Pernambuco, Recife, Brasil;; b Curso de Farmácia, Faculdade Pernambucana de Saúde, Recife, Brasil;; c Centro de Pesquisa Aggeu Magalhães, Recife, Brasil;; d Programa de Pós-graduação, Universidade CEUMA, Sao Luis, Brasil;; e Departamento de Antibióticos, Universidade Federal de Pernambuco, Recife, Brasil

**Keywords:** Antifungal agents, natural products, Caatinga biome

## Abstract

**Context:** *Buchenavia tetraphylla* (Aubl.) RA Howard (Combretaceae: Combretoideae) is an ethnomedicinal plant with reported antifungal action.

**Objective:** This study evaluates the antimicrobial activity of *B. tetraphylla* leaf extracts against clinical isolates of *Candida albicans*. The morphological alterations, combinatory effects with fluconazole and the cytotoxicity of the active extract were analyzed.

**Materials and methods:** Extracts were obtained using different solvents (hexane: BTHE; chloroform: BTCE; ethyl acetate: BTEE; and methanol: BTME). Antimicrobial activity was determined by the broth microdilution method using nine strains of *C. albicans* isolated from vaginal secretions and one standard strain (UFPEDA 1007).

**Results:** All extracts showed anti-*C. albicans* activity, including against the azole-resistant strains. The MIC values ranged from 156 to 2500 μg/mL for the BTHE; 156 to 1250 μg/mL for the BTCE; 625 to 1250 μg/mL for the BTME and 625 μg/mL to 2500 μg/mL for the BTEE. BTME showed the best anti-*C. albicans* activity. This extract demonstrated additive/synergistic interactions with fluconazole. Scanning electron microscopy analysis suggested that the BTME interferes with the cell division and development of *C. albicans*. BTME showed IC_50_ values of 981 and 3935 μg/mL, against J774 macrophages and human erythrocytes, respectively. This extract also enhanced the production of nitric oxide by J774 macrophages.

**Discussion and conclusion:** *Buchenavia tetraphylla* methanolic extract (BTME) is a great source of antimicrobial compounds that are able to enhance the action of fluconazole against different *C. albicans* strains; this action seems related to inhibition of cell division.

## Introduction

*Candida albicans* is a polymorphic fungus recognized as one of the most common fungal pathogens, responsible for a range of diseases (Mayer et al. 2013). This yeast is usually found in the normal microbiota of several distinct anatomical sites such as skin, mouth and genitourinary and digestive systems (Pfaller & Diekema 2010; Gulati & Nobile 2016). However, some adverse environmental conditions such as pH shift, nutritional deficiency, use of antibiotics or variations in the immune status of the host, can enable this microorganism to cause infections, which range from superficial dermal and mucosal cases to more severe systemic infections (candidemia and invasive candidiasis, frequently associated with patients with compromised immunity). Several factors, related to both host and pathogen, are recognized as crucial for infection development; for example, *C. albicans* possesses a range of virulence factors, associated to adherence, filamentation, biofilm formation and secretion of proteases. Additionally, antibiotic and immunosuppressive therapies, diseases (human immunodeficiency virus, diabetes), and aging are also factors that favour *C. albicans* infections (Gow & Hube 2012; Mayer et al. 2013; Fadda et al. 2015).

The treatment of *C. albicans* infection is based on the use of systemic antifungals (fluconazole or itraconazole) and topical nystatin (Pappas et al. 2015). An increase in the resistance of *C. albicans* to antifungals has been observed in recent decades, which makes this yeast a serious concern for the global health system. Indeed, the development of new and effective approaches to treat fungal infections remains one of the major challenges for modern medicine (Sardi et al. 2013).

Traditional medicinal plants from underexploited environments, such as the Caatinga (semi-arid region), an exclusively Brazilian biome, have been the subject of various studies in the search for new antimicrobial compounds, due to their exceptional activity against microorganisms (Oliveira et al. 2012; Silva et al. 2014). *Buchenavia tetraphylla* (Aubl.) RA Howard (Combretaceae: Combretoideae) is a Neotropical plant, distributed from Cuba (Caribbean) to Brazil (South America) (Weaver 1991). In Brazil, this plant is popularly known as ‘Tanimbuca’ and is listed as an ethnomedicinal plant used by the traditional communities of northeastern Brazil (Agra et al. 2007, 2008). Our group has shown that extracts and fractions of *B. tetraphylla* leaves have a broad-spectrum of antimicrobial activities, inhibiting both Gram-positive and Gram-negative bacteria and fungi (including *C. albicans*) (Oliveira et al. 2012). Additionally, a recent study has shown that *B. tomentosa* had a potential antifungal effect against different *Candida* species (Teodoro et al. 2015). In this context, this study aimed to evaluate the antimicrobial activity of *B. tetraphylla* extracts alone or in combination with fluconazole against clinical isolates of *C. albicans* isolated from vaginal secretions, and to analyze the ultrastructural changes and the combinatory effect with fluconazole induced by the active extract.

## Materials and methods

### Plant material: collection, identification and extraction

Leaves of *B. tetraphylla* were collected in the *Parque Nacional do Catimbau* (PARNA do Catimbau, Pernambuco, Brazil) in November of 2013. The taxonomic identification was performed by Dr Alexandre Gomes da Silva, at the Herbarium of the Instituto Agronômico de Pernambuco (IPA), where the voucher specimen (Number 84.104) was deposited. Leaves were dried using an incubator at 45 °C for 2 to 3 days. The dried material was ground into powder form using a grinder followed by a Waring blender.

For extraction, dried leaves (25 g) were mixed with hexane (100 mL) in a shaker at 125 rpm for 72 h at 25 °C. Then, the extract was filtered and the supernatant was concentrated in a rotary vacuum. The residue was suspended in 100 mL of chloroform. The extraction procedure was repeated and the residue was resuspended in 100 mL of ethyl acetate. Finally, the residue was subjected to methanol extraction. All extract samples were diluted in 100% dimethyl sulfoxide and stored at 4 °C prior to use.

### Phytochemical analysis

The extracts from *B. tetraphylla* were submitted to thin-layer chromatography (TLC) assays in order to perform a qualitative detection of their functional compounds. The extracts were loaded in silica gel F254 + 366 plates (20 × 20 cm; <AQ5>), and developed in a solvent system: A (toluene/dioxan/acetic acid, 180:45:5, v/v) and B (hexane/diethyl ether/formic acid, 130:80:20, v/v). Bands were revealed under UV light (254/365 nm), using ceric sulphate and ferric chloride. The bands were identified by their Rf values and compared with the standards used.

### Antimicrobial assays

#### *Candida albicans* strains

A total of 10 *C. albicans* strains were used in this study. The standard strain (UFPEDA1007) was obtained from the Microbial Collection of the *Departamento de Antibióticos, Universidade Federal de Pernambuco* (UFPEDA). The clinical strains were isolated from vaginal secretions at different clinical laboratories in Recife (Pernambuco, Brazil) and kindly provided by them between September and December 2012.

The antibiotic-susceptibility profile of each isolate was performed according to the CLSI recommendations (CLSI 2011) using the disc-diffusion assay on Sabouraud dextrose agar (SDA). In brief, colonies from overnight cultures of *C. albicans* were suspended in sterile saline water equivalent to a 0.5 McFarland standard. The suspension (100 μL) was spread over an SDA plate and the antibiotic disc was applied aseptically onto the surface. Afterwards, the plates were incubated at 30 °C for a period of 48 h and then each inhibition diameter zone (IDZ) was measured and interpreted as shown in Table 1. The antibiotics used were amphotericin B, fluconazole, itraconazole and ketoconazole.

**Table 1.** Antibiotic-resistance profile of *C. albicans* clinical strains isolated from vaginal secretion samples.

Strain	Amphotericin B	Ketoconazole	Fluconazole	Itraconazole
F01	R	S	S	S
F02	S	S	R	R
F03	S	S	S	S
F08	S	S	R	R
F11	S	S	R	R
F14	S	S	I	I
F22	S	S	I	I
F23	S	S	S	S
F27	S	S	I	I
Interpretation of IDZ values (mm) (CLSI, 2011)				
Drugs		Sensitive (S)	Intermediate (I)	Resistant (R)
Amphotericin B		≥15	14–10	<10
Ketoconazole		≥28	27–21	≤20
Fluconazole		≥19	18–15	≤15
Itraconazole		≥23	22–14	<13

The multiple antibiotic resistance (MAR) index was calculated as previously described by Krumperman (1983) using the formula MAR = *x*/*y*, where ‘*x*’ is the number of antibiotics to which the isolate demonstrated resistance; and ‘*y*’ is the total number of antibiotics tested.

### Determination of minimal inhibitory concentration (MIC) and minimum fungicidal concentration (MFC)

The minimal inhibitory concentration (MIC) was determined by the microdilution method (CLSI 2011). Twofold serial dilution of each extract was prepared in Sabouraud broth and 10 μL of yeast suspension (approximately 1.5 × 10^8^ CFU/mL) were added. The microplates were incubated at 30 °C. After 48 h, 50 μL of resazurin solution (0.01%) were added to each well. The plates were re-incubated for 2 h at 30 °C, and any colour changes from purple to pink was recorded as microbial growth. The lowest concentration at which no colour change occurred was taken as the MIC. Afterwards, cultures were seeded in SDA plates and incubated for 48 h at 30 °C to determine the minimum fungicidal concentration (MFC).

### Combinatory effects of *B. tetraphylla* methanolic extract and fluconazole

The combinatory effects of the BTME and fluconazole were evaluated against five *C. albicans* strains (F03, F08, F11, F22, 1007). Both samples were added to microplates containing SAB and a twofold serial dilution was prepared (5000 to 98 μg/mL for BTME and 100 to 0.2 μg/mL for fluconazole). The antimicrobial action was evaluated as described for MIC, and after 48 h, the drug/extract interaction was assessed algebraically by determining the Fractional Inhibitory Concentration indices (ΣFIC), according to the following equation:

$$\Sigma \text{FIC }=\;\left({\text{MIC}}_{\text{E}}+\text{F}/{\text{MIC}}_{\text{E}}\right)\;+\;\left({\text{MIC}}_{\text{F}}+\text{E}/{\text{MIC}}_{\text{F}}\right)$$

MIC_E_ or MIC_F_: the MIC extract or MIC fluconazole; MIC_F+D_: the MIC_E_ when in combination with fluconazole; MIC_D+F_: the MIC of fluconazole when in combination with extract; Data interpretation: ΣFIC ≤0.5: synergism (syn); 0.5 < ΣFIC ≤1: addition (add); 1 < ΣFIC < 4: non-interaction (non); ΣFIC ≥4: antagonism (ant) (Vuuren & Viljoen 2011).

### Scanning electron microscopy (SEM)

To evaluate the morphological changes induced by the BTME extract, a sample of 100 μL of a diluted overnight culture of *C. albicans* (approximately 1.5 × 10^8^ CFU/mL) was added to 1 mL of SAB and mixed with the BTME (MIC). After incubation (12 h at 30 °C) the cells were treated as described by da Silva et al. (2013) and imaged with a Quanta 200 F scanning electron microscope (FEI company).

### Determination of nitric oxide production and cell viability in macrophages

J774 macrophages (1 × 10^5^ cells/mL) were seeded in a 96-wells plate for 24 h at 37 °C and 5% CO_2_ and then treated with the BTME (156–2500 μg/mL) for another 24 h. After the treatment, the supernatant was used to determine nitric oxide (NO) production, and the adherent cells were used for viability assays. For NO determination, the supernatant (100 μL) from each well was mixed with 100 μL of Griess reagent in a 96-wells plate. After incubation for 15 min at room temperature, the optical density was determined at 540 nm with a microplate reader (Benchmark plus, Bio-Rad, Hercules, CA). The nitrite content (μM/10^6^ cells) was quantified by extrapolation from sodium nitrite standard curve in each experiment. Cell viability was evaluated using the MTT assay, which measures the metabolic conversion of the 3-(4,5-dimethylthiazol-2-yl)-2,5-diphenyltetrazolium bromide (MTT) salt by mitochondria of viable cells, by the use of coloured formazan dye. At the end of the incubation, the medium was removed and an MTT solution (5000 μg/mL in RPMI) was added to the cells that were further incubated for 3 h. Afterwards, the medium was removed and the intracellular formazan product was dissolved in DMSO. Optical density (OD) was measured at 595 nm. Cell viability was calculated in comparison to the OD obtained by untreated cells.

### Haemolytic assay

Blood (5–10 mL) samples were obtained from healthy volunteers by venipuncture and placed in heparinized tubes, after written informed consent was obtained. Human erythrocytes were isolated by centrifugation (1500 rpm for 10 min) and washed three times with phosphate-buffered saline (PBS; pH 7.4). Each test tube received 1.1 mL of erythrocyte suspension (1%) and 0.4 mL of the various extracts at different concentrations (156–2500 μg/mL). Cells incubated in PBS or in a solution of saponin from *Quillaja* sp. (0.0025%) were used as negative and positive controls for haemolytic activity, respectively. After 60 min of incubation, cells were centrifuged and the absorbance of supernatant was read at 540 nm. The haemolytic activity was expressed by the following formula:

Haemolytic activity (%)=(As - Ab)×100/(Ac - Ab)

*A*b = solvent absorbance, *A*s = sample absorbance; *A*c =saponin absorbance.

### Statistical analysis

All experiments were performed in quadruplicate with at least two independent experiments. Results are expressed as mean ± standard deviation (SD). Statistical analyses were performed by Student's *t* test. All analyses were carried out using the Statistica 8.0 software. Differences were considered significant at *p* < 0.05. The correlation indices were calculated using the Pearson coefficient (*ρ*).

## Results

### Phytochemical analysis

The phytochemical profile of extracts from leaves of *B. tetraphylla* is shown in Table 2. The results showed that all extracts have at least one class of compound reported as an antimicrobial agent. The hexane extract (BTHE) presented hydrocarbons, glycosides, sugar and terpenes. Flavonoids, terpenes and tannins were detected in the ethyl acetate extract (BTEE), while only terpenes and sugar were found in the chloroform extract (BTCE). Finally, flavonoids and tannins were detected in the methanol extract (BTME).

**Table 2.** Phytochemical analysis of *B. tetraphylla* leaves extracts.

	Extract			
Phytochemical compounds	BTHE	BTEE	BTCE	BTME
Hydrocarbons	+	+	–	–
Sugars	+	–	+	–
Glycosides	+	–	–	–
Terpenes	+	+	+	–
Flavonoids	–	+	–	+
Tannins	–	+	–	+

BTHE: *Buchenavia tetraphylla* extracts hexane; BTEE: *B. tetraphylla* extracts Ethyl acetate; BTCE: *B. tetraphylla* extracts Chloroform; BTME: *B. tetraphylla* extracts Methanol.

### Antibiotic susceptibility of *C. albicans* strains

All clinical isolates of *C. albicans* tested in this study had their antibiotic-susceptibility profile analyzed (Table 1). According to the disc-diffusion assay, two strains (F03 and F23) were susceptible to all tested antifungal agents; another three isolates (F14, F23 and F27) were susceptible to amphotericin B and ketoconazole and showed intermediate susceptibility to fluconazole and itraconazole; the strain F01 was only resistant to amphotericin B; finally, the strains F02, F08 and F11 showed resistance to fluconazole with cross-resistance to itraconazole (MAR index of 0.5). Fluconazole resistance of each strain was confirmed by MIC determination as recommend by CLSI (CLSI 2011). Strains F03, F08 and 1007 were inhibited by fluconazole at 3.125 μg/mL, while strains F11 and F22 were inhibited by the same drug at 6.25 μg/mL.

### Anti-*C. albicans* action of *B. tetraphylla* extracts

The inhibitory action of *B. tetraphylla* extracts was evaluated against 10 *C. albicans* strains (Table 3). The extracts showed anti-*C. albicans* activity against all tested strains, including the azole-resistant strains (F02, F08 and F11). The MIC values ranged from 156 to 2500 μg/mL for the BTHE; 156 to 1250 μg/mL for the BTCE; 625 to 1250 μg/mL for the BTME and 625 μg/mL to 2500 μg/mL for the BTEE. The BTME and BTCE showed the best antimicrobial potential with no significant difference between their average MFC values (*p* > 0.05). A moderate negative correlation was observed between the MAR indexes of all strains and MIC (*ρ* = −0.39) or MFC (*ρ* = −0.33) values of the BTME, indicating this extract was slightly more active against the resistant strains. Moderate and weak positive correlations were found between the MAR indexes and MIC (*ρ* = 0.51) or MFC (*ρ* = 0.14) values of the BTCE. Additionally, the MFC/MIC ratios ranged from 2 to 4 for the BTME, thus it was a fungicidal agent against all tested strains. Both fungicidal and fungistatic effects were observed for the BTCE (MFC/MIC ratios ranged from 2 to 8), but fungicidal action was more prominent (for nine strains). Since the BTME showed the strongest activity it was chosen for further biological activity assays.

**Table 3.** The anti-*Candida albicans* activity of extracts from *B. tetraphylla* leaves.

		*B. tetraphylla* extracts											
		Ethyl acetate			Chloroform			Hexane			Methanol		
*C. albicans strains*	MAR index	MIC^a^	MFC^a^	MFC/MIC	MIC^a^	MFC^a^	MFC/MIC	MIC^a^	MFC^a^	MFC/MIC	MIC^a^	MFC^a^	MFC/MIC
F01	0.25	1250	>5000	>4	625	5000	8	625	1250	2	625	1250	2
F02	0.5	1250	5000	4	1250	2500	2	2500	5000	2	625	2500	4
F03	0	2500	>5000	>2	625	2500	4	625	2500	4	1250	2500	2
F08	0.5	625	>5000	>8	625	1250	2	1250	5000	4	625	1250	2
F11	0.5	1250	2500	2	625	1250	2	156	1250	8	625	1250	2
F14	0	625	5000	8	625	1250	2	625	>5000	>8	625	1250	2
F22	0	1250	5000	4	625	2500	4	1250	2500	2	625	2500	4
F23	0	2500	5000	2	625	1250	2	1250	2500	2	1250	2500	2
F27	0	625	1250	2	625	1250	2	1250	2500	2	625	2500	4
UFPEDA 1007	0	625	2500	4	156	312	2	1250	5000	4	625	1250	2
Average MIC		1250 ± 721 μg/mL			641 ± 260 μg/mL			1078 ± 636 μg/mL			750 ± 264 μg/mL		
Average MFC		4125 ± 1450 μg/mL			1096 ± 1305 μg/mL			3250 ± 1581 μg/mL			1875 ± 659 μg/mL		

a MIC e MMC is expressed at μg/mL; MAR index: multiple antibiotic resistance index; UFPEDA: Microbial Collection of the *Departamento de Antibióticos*, *Universidade Federal de Pernambuco*.

### Combinatory effects of the BTME and fluconazole

**Figure 1.**
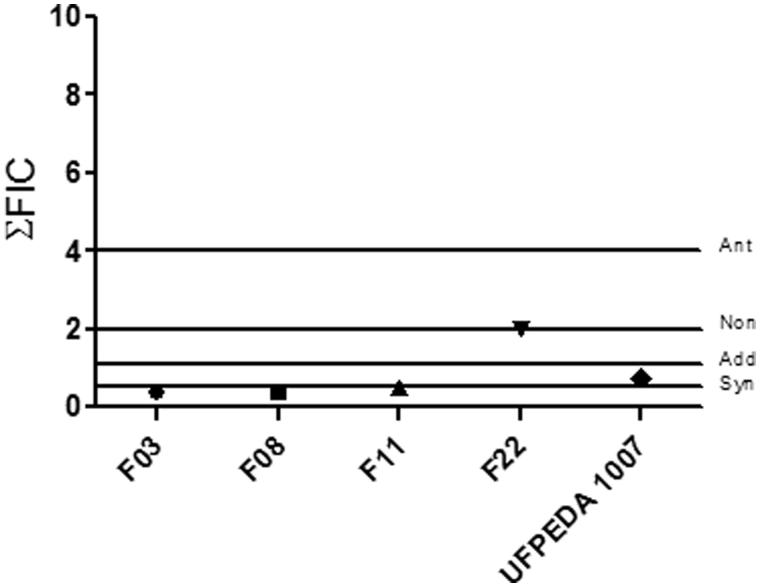
Combinatory effects of BTME and fluconazole against *Candida albicans*. non: non-interactive effect; add: additive effect; syn: synergistic effect; ant: antagonistic effect.

The combinatory effects of the BTME and fluconazole are shown in Figure 1. The extract had the ability to increase the action of fluconazole in most strains through additive (20% of strains; ΣFIC =0.75) or synergetic (60% of strains; ΣFIC values of 0.375 and 0.5) effects. Non-interaction was observed against one strain (ΣFIC =2).

### Morphological changes induced by the BTME

**Figure 2.**
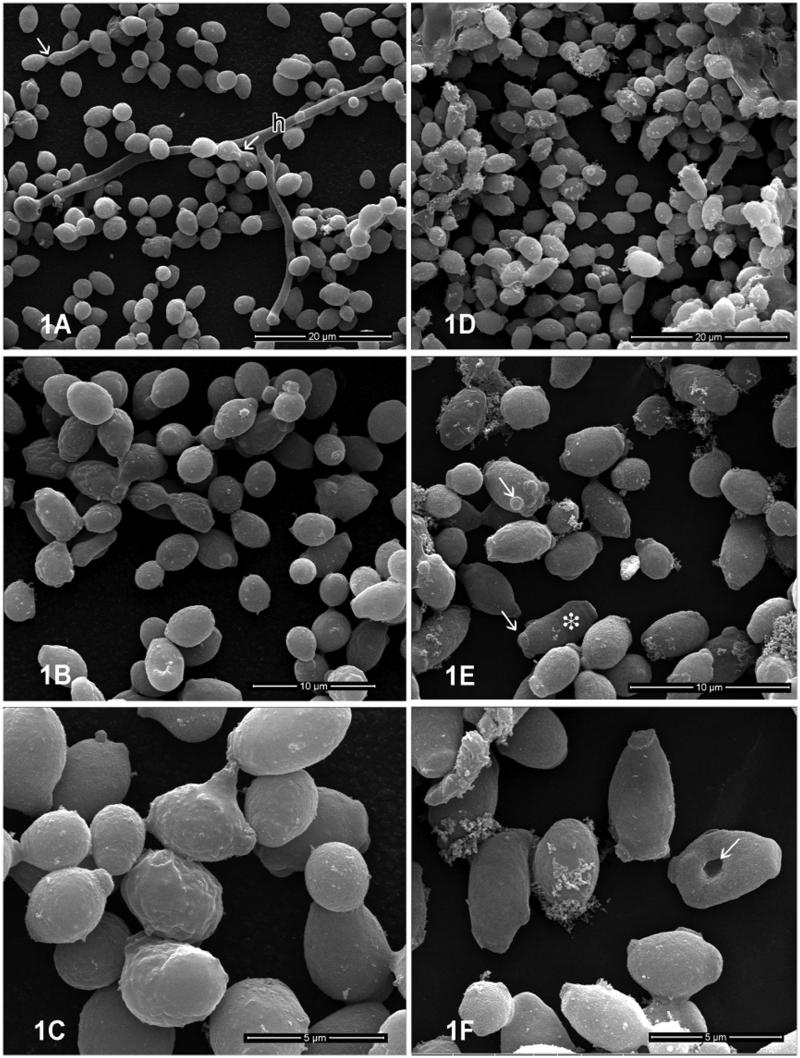
Effects of BTME on *C. albicans*. (a–c) Control cells; (d–f) cells treated with BTME at MIC for 12 h. (a–c) Ultrastructural aspects of untreated *C. albicans* culture at low (a) and high magnification (b–c) showing the presence of single and budding yeast cells with evident fragile blastoconidial septum (white arrow). (h) True hyphal structures could be also observed. Treated *C. albicans* culture at (d) low and (e–f) high magnifications. Note in (e) the presence of elongated cells (white asterisk) with multiple scars (white arrow). (f) Cells presenting surface depressions are indicated in white arrow.

The morphological alterations induced by the BTME in the fluconazole-resistant F08 strain were evaluated using scanning electron microscopy. This strain was chosen because it presents a high degree of cell surface hydrophobicity *in vitro* (data not shown), a property related to the increase of *C. albicans* adherence and virulence (Blanco et al., 2009; Silva-Dias et al. 2015). After 12 h of cultivation, untreated samples (Figures 2(a–c)) showed a predominance of budding blastoconidial cells with a normal elliptical shape, smooth surface and formation of hyphal structures exhibiting a homogeneous, elongated shape without constrictions (Figure 2(a)). BTME-treated cells (Figures 2(d–f)) showed intense deposition of flocculated material on the cell surface, more elongated blastoconidial morphology with several scars (Figure 2(e)). However, no filamentation could be observed and hyphal structures were not seen in treated samples. Cells presenting depressions on the cell surface could also be observed (Figure 2(f)). No ruptured cells or alterations on the yeast cell surface were detected.

### Effects of the BTME on cell viability and NO production

**Figure 3.**
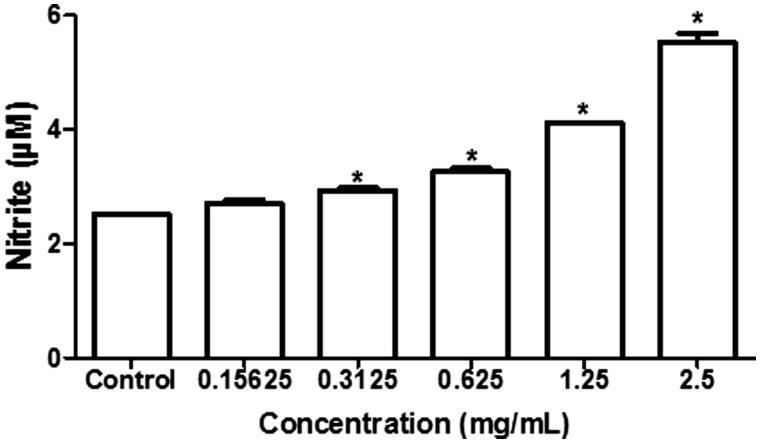
Effect of BTME on nitric oxide production by J774 macrophages. *Significant differences in relation to control (*p* < 0.05).

The treatment of J774 macrophages with different concentrations of the BTME resulted in significant production of NO at concentrations from 312.5 to 2500 μg/mL (*p* > 0.05), in a dose-dependent manner (Figure 3). Regarding cytotoxicity, the BTME extract showed IC_50_ values of 981 μg/mL against J774 macrophages and 3935 μg/mL against erythrocytes.

## Discussion

*C. albicans* is the most prevalent pathogen associated with vulvovaginal candidiasis, and it is estimated that it affects approximately 75% of women at least once during their lifetime. This pathology represents significant health issues for women of childbearing age, as well as diabetic and immuno-deficient patients. Vulvovaginal candidiasis is associated with considerable morbidity, increased healthcare costs, distress, pain and sexual dysfunction (Cassone 2014). *C. albicans* strains isolated from vaginal secretions have shown increased resistance towards antifungal agents, especially fluconazole. It is important to highlight that the breakpoint of *in vitro* susceptibility for strains isolated from vaginal secretions has been suggested to be 1 μg/mL instead of 8 μg/mL, this is due to the specific pharmacokinetic characteristics of the vagina (Sobel et al. 2003; Marchaim et al. 2012). In this context, this work evaluated the antifungal action of extracts from leaves of *B. tetraphylla* against fluconazole-resistant and fluconazole-sensitive strains of *C. albicans* isolated from human vaginal secretions.

It has been reported that *B. tetraphylla* leaves are a source of compounds with anti-*C. albicans* activity (Oliveira et al. 2012). Furthermore, Teodoro et al. (2015) found remarkable anti-*Candida* activity of another member of the *Buchenavia* genus, *B. tomentosa*. All the extracts were also effective against the clinical isolates tested. Furthermore, the extraction method used in this present paper increased the antifungal potential of this plant, as the average MIC values of the standard strain were up to 40 times lower than those reported by Oliveira et al. (2012). Among the extracts, the BTME and BTCE showed the best activity and no significant differences were observed between their MIC or MFC values. The BTME is more effective against the fluconazole-resistant strains than the BTCE, as confirmed by correlation analysis (the Pearson coefficient for their MIC values was −0.032; and 0.076 for their MFC values). Two mammalian cell types were used to determine the cytotoxic potential of each extract: human erythrocytes and mice macrophages. Both models are widely used to assess the toxicity of plant-derived products (Oliveira et al. 2012; Santos Aliança et al. 2014; Sant’Anna da Silva et al. 2016). In the cytotoxicity assays, the BTME showed IC_50_ values of 981 and 3935 μg/mL against J774 macrophages and human erythrocytes, respectively. These values are higher than the average MIC of the BTME (625 μg/mL). Therefore, it was decided to evaluate the morphological changes induced by the BTME and its effects when in combination with fluconazole.

Fluconazole is recommended as the first choice for treating pathologies caused by *C. albicans*, mainly due to its high efficiency and good pharmacokinetic properties (Pappas et al. 2015). However, the appearance of fluconazole resistance represents an enormous limitation for its use in the treatment of candidiasis (Morschhäuser 2016). Consequently, combination therapy has been indicated as a useful alternative to manage *Candida* infections (Cui et al. 2015). Measurement of the combinatory effects of natural products and antimicrobial agents has been taken as a new strategy in the battle against drug resistance (Hemaiswarya et al. 2008; Wagner & Ulrich-Merzenich 2009; Cui et al. 2015) and a range of plant-derived products have been used to increase or restore the action of some antifungal agents such as fluconazole (Khan & Ahmad 2012; Avijgan et al. 2014; da Silva et al. 2014). The BTME was able to increase fluconazole activity against the five resistant strains by addictive and synergetic effects. Compounds from both flavonoid and tannin classes (same classes detected in the BTME) are reported as enhancers of fluconazole activity (Endo et al. 2010, da Silva et al. 2014).

Depending on environmental conditions, *C. albicans* can assume three different morphotypes during its development: blastoconidial, pseudohyphal and hyphal forms. Furthermore, *C. albicans* easily develops into a biofilm on surfaces, which when compared to planktonic cells, displays stronger resistance to a wide variety of antifungal agents (Staniszewska et al. 2012; Ma et al. 2015). Different from the control cells, morphological observation showed a predominance of single, isolated blastoconidia over the budding cells and no true hyphal development. These results suggest that the BTME substantially affected the morphogenetic transition of *C. albicans*, which is crucial to its virulence (Staniszewska et al. 2012). The ability to produce hyphae (and them switch between yeast and filamentous forms) is of extreme importance to the pathogenesis of candidiasis and is considered the central virulence attribute (Berman & Sudbery 2002; Dalle et al. 2010; Mayer et al. 2013). The yeast-to-hyphae transition is related to tissue damage and invasion and the host inflammatory response, as well as mediating *Staphylococcus aureus* co-infection (Peters et al. 2014; Schlecht et al. 2015). The ability of the BTME to inhibit hyphal formation can be considered an attractive characteristic for anti-*C. albicans* therapy, especially during biofilm formation (Jacobsen et al. 2012).

Finally, the BTME induced the significant production of NO from macrophages. NO is a reactive oxygen nitrogen species which is recognized as a marker of macrophage activation, playing an essential role in antimicrobial defence. Specifically, NO suppression is a mechanism used by *C. albicans* to evade the immune system response, thus macrophage function modulation is a potential aim for antifungal therapy (Collette et al. 2014).

In conclusion, this study demonstrated that *B. tetraphylla* is a great source of antimicrobial compounds able to enhance the action of fluconazole against different *C. albicans* strains. The methanol extract induced significant morphological changes in treated cells and enhanced NO production *in vitro*. Therefore, it is presented as a promising source of antifungal agent(s) for the treatment of *C. albicans*. The identification, molecular and *in vivo* studies of the active substance(s) will be areas of important future research aimed at finding alternative, new strategies for controlling *C. albicans* infections using natural products from the Caatinga.
